# Novel Insights into the Protective Properties of ACTH_(4-7)_PGP (Semax) Peptide at the Transcriptome Level Following Cerebral Ischaemia–Reperfusion in Rats

**DOI:** 10.3390/genes11060681

**Published:** 2020-06-22

**Authors:** Ivan B. Filippenkov, Vasily V. Stavchansky, Alina E. Denisova, Vadim V. Yuzhakov, Larisa E. Sevan’kaeva, Olga Y. Sudarkina, Veronika G. Dmitrieva, Leonid V. Gubsky, Nikolai F. Myasoedov, Svetlana A. Limborska, Lyudmila V. Dergunova

**Affiliations:** 1 Institute of Molecular Genetics, Russian Academy of Sciences, 123182 Moscow, Russia; bacbac@yandex.ru (V.V.S.); sudarolg@img.ras.ru (O.Y.S.); veronuska@mail.ru (V.G.D.); nfm@img.ras.ru (N.F.M.); limbor@img.ras.ru (S.A.L.); lvdergunova@mail.ru (L.V.D.); 2 Pirogov Russian National Research Medical University, Federal Center of Cerebrovascular Pathology and Stroke, Ministry of Health Care of Russian Federation, 117342 Moscow, Russia; dalina543@gmail.com (A.E.D.); gubskii@mail.ru (L.V.G.); 3 A. Tsyb Medical Radiological Research Center–Branch of the National Medical Research Radiological Center of the Ministry of Health of the Russian Federation, 249031 Obninsk, Russia; yuzh_vad@mail.ru (V.V.Y.); larisa.sevankaeva@mail.ru (L.E.S.)

**Keywords:** tMCAO, mRNA expression, RNA-Seq, synthetic melanocortin derivative ACTH_(4-7)_PGP (Semax), peptide regulation

## Abstract

Cerebral ischaemia is the most common cause of impaired brain function. Biologically active peptides represent potential drugs for reducing the damage that occurs after ischaemia. The synthetic melanocortin derivative, ACTH_(4-7)_PGP (Semax), has been used successfully in the treatment of patients with severe impairment of cerebral blood circulation. However, its molecular mechanisms of action within the brain are not yet fully understood. Previously, we used the transient middle cerebral artery occlusion (tMCAO) model to study the damaging effects of ischaemia–reperfusion on the brain transcriptome in rats. Here, using RNA-Seq analysis, we investigated the protective properties of the Semax peptide at the transcriptome level under tMCAO conditions. We have identified 394 differentially expressed genes (DEGs) (>1.5-fold change) in the brains of rats at 24 h after tMCAO treated with Semax relative to saline. Following tMCAO, we found that Semax suppressed the expression of genes related to inflammatory processes and activated the expression of genes related to neurotransmission. In contrast, ischaemia–reperfusion alone activated the expression of inflammation-related genes and suppressed the expression of neurotransmission-related genes. Therefore, the neuroprotective action of Semax may be associated with a compensation of mRNA expression patterns that are disrupted during ischaemia–reperfusion conditions.

## 1. Introduction

Focal cerebral ischaemia or stroke is one of the leading causes of mortality and disability in developed countries. The development of effective strategies to treat ischaemic stroke is an important issue in modern medicine and pharmacology. A drug currently found to be efficient in cerebral stroke therapy is a synthetic neuroprotective peptide called Semax (Met-Glu-His-Phe-Pro-Gly-Pro). It bears a fragment of adrenocorticotropic hormone 4-7 (Met-Glu-His-Phe) and the C-terminal tripeptide Pro-Gly-Pro (PGP), last one was included to ensure the resistance of Semax to peptidases. The neuroprotective and neurotrophic effects of Semax are reliably established [1,2,3,4,5,6,7,8]. It has been shown that Semax improves cognitive function in both rodents and humans. The peptide facilitates acquisition of food-motivated and passive-avoidance tasks in healthy animals and has a protective effect in a model of stress-induced memory impairment in rats [9,10,11]. In experimental models of cerebral ischaemia Semax administration led to the recovery of the animals’ ability to learn in a Morris water maze and passive-avoidance task [12,13]. A clinical study has shown the efficacy of Semax in the treatment of patients with ischaemic stroke. The peptide improves functional recovery and motor performance [14,15]. Nevertheless, the molecular mechanisms of its neuroprotective effects remain unclear.

Transcriptome analysis is one of the approaches used to study the functioning of the brain in ischaemia and under the action of drugs. To study the effect of drugs on the genes functioning under cerebral ischaemia conditions, models of cerebral ischaemia in laboratory animals have been widely used [16,17,18,19,20,21,22]. Previously, using models of global incomplete cerebral ischaemia we have shown the effect of Semax on the expression of the limited number of genes that encode neurotrophic factors and their receptors [23,24,25]. Using microchips RatRef-12 BeadChips, it was shown that peptide Semax affects the expression of genes related to the immune and vascular systems in rat fronto-parietal cortex after permanent middle cerebral artery occlusion (pMCAO) [26,27]. At present, to study the molecular mechanisms of the effects of Semax, we used another model of transient middle cerebral artery occlusion (tMCAO). This model reflects events that occur in ischaemic stroke in humans after treatment with thrombolytic agents [28,29]. The results of clinical studies indicate that, currently, thrombolysis is one of the most effective and affordable methods of treatment of ischaemic stroke [30,31]. In the tMCAO model conditions, which were based on endovascular artery occlusion (90 min) and subsequent reperfusion, we identified hundreds of differentially expressed genes (DEGs) [32]. In particular, we revealed the activation of a large number of genes involved in inflammation, the immune response, apoptosis and the stress response. Simultaneously, a massive downregulation of genes that ensure the functioning of neurotransmitter systems was observed in tMCAO conditions [32]. We studied genome-wide gene transcription of the subcortex tissue using the tMCAO model in an attempt to research the mechanisms of the neuroprotective effects of Semax. This study allowed us to observe alterations to the transcriptome profile and to reveal previously unknown compensation effects of Semax on the biological processes and signal pathways, which apparently provides the neuroprotective effects of the peptide in ischaemia–reperfusion (IR) conditions.

## 2. Materials and Methods

### 2.1. Animals

White 2-month-old male rats of the Wistar line (weight, 200–250 g) were obtained from the Experimental Radiology sector in A. Tsyb Medical Radiological Research Center, Obninsk, Russian Federation. The animals were maintained on a 12 h light/dark cycle at a temperature of 22–24 °C, with free access to food and water. The animals were divided into the “ischaemia–reperfusion” (IR) and “ischaemia–reperfusion after Semax administration” (IS) groups.

### 2.2. Transient Cerebral Ischaemia Rat Model

#### 2.2.1. Operation

The transient cerebral ischaemia rat model was induced by endovascular occlusion of the right middle cerebral artery using a monofilament (Doccol Corporation, Sharon, MA, USA) for 90 min [33]. The rats were decapitated at 4.5 or 24 h after tMCAO (group IR—“IR4.5” or “IR24”, respectively and group IS—“IS4.5” or “IS24”, respectively). Prior to the surgical procedure, rats were anaesthetized using 3% isoflurane; the anaesthesia was maintained using 1.5–2% isoflurane and the EZ–7000 small animal anaesthesia system (E-Z Anaesthesia, Braintree, MA, USA). The sham-operated rats (SH) were subjected to a similar surgical procedure under anaesthesia (neck incision and separation of the bifurcation), but without tMCAO.

#### 2.2.2. Semax Administration

To rats of the IS group, the neuropeptide Semax was administered intraperitoneally at a dose of 10 μg/100 g rat weight as previously described [11,26,27,34] at 90 min, as well as at 2.5 and 6.5 h after tMCAO. To rats of the IR groups, saline was administered intraperitoneally at 90 min, as well as at 2.5 and 6.5 h after the surgical procedure.

### 2.3. Magnetic Resonance Imaging

The magnetic resonance imaging (MRI) study of the characteristics and size of the ischaemic injury of rat brains was carried out using a small animal 7T system from ClinScan tomograph (Bruker BioSpin, Billerica, MA, USA). The standard protocol included the following modes: diffusion-weighted imaging (DWI) with mapping of the apparent diffusion coefficient (ADC) for assessing acute ischaemic damage (TR/TE = 9000/33 ms; b factors = 0 and 1000 s/mm^2^; diffusion directions = 6; averages = 3; spectral fat saturation; FOV = 30 × 19.5 mm; slice thickness = 1.0 mm; matrix size = 86 × 56), and T2–weighted imaging (T2 WI) in the transverse plane (Turbo Spin Echo with restore magnetization pulse; turbo factor = 10; TR/TE = 5230/46 ms; averages = 2; spectral fat saturation; FOV = 30 × 21.1 mm; slice thickness = 0.7 mm; matrix size = 256 × 162). Three-dimensional time-of-flight magnetic resonance angiography (3D-TOF MRA) was used for visualization of the main arteries and control of the recanalization (3D Gradient Echo with RF spoiling and flow compensation; TR/TE = 30/4.55 ms; slabs = 4; flip angle = 70; averages = 1; FOV = 35 × 19.3 mm; slice thickness = 0.2 mm; matrix size = 320 × 176). A quantitative assessment of the volume of the infarction focus was performed using the ImageJ software package (Wayne Rasband, National Institute of Mental Health, Bethesda, MD, USA). MRI was performed immediately before decapitation in rats from the IR4.5 and IS4.5 groups. In rats from the IR24 and IS24 groups, MRI was performed twice: at 4.5 h after tMCAO and immediately before decapitation.

### 2.4. Histological Examination of Rat Brains

Tissue samples of rat brains at 24 h after occlusion (*n* = 4) and after sham operation (*n* = 4) were immersed in Bouin’s fluid for 24 h and washed with 70% ethanol. The tissue samples were dehydrated and embedded in Histomix® (BioVitrum, St. Petersburg, Russia). Tissue sectioning was performed with the orientation of two tissue blocks for subsequent excision into coronary sections at the level from −4.0 to −0.5 and from −0.5 to +5 mm from the bregma. Sections with a thickness of 5–6 μm obtained through 0.5–1 mm on a microtome (Leica RM2235, Wetzlar, Germany) were stained with haematoxylin and eosin (H&E staining) and toluidine blue in Nissl modifications (BioVitrum, St. Petersburg, Russia) after dewaxing. Histological specimens were examined under a microscope (Leica DM 1000, Wetzlar, Germany) with a micrograph to digital camera (Leica ICC50 HD, Wetzlar, Germany). Morphological analysis was performed with allowance for normal and pathological central nervous system cells variants [35,36,37]. Stereotactic mapping of the damaged zones and accurate determination of the level of sections were performed according to an atlas of the rat brain [38].

### 2.5. RNA Isolation

Tissues were placed in RNAlater solution for 24 h at 0 °C and then stored at −70 °C. Total RNA from the subcortex, was isolated using TRIzol reagent (Invitrogen, Thermo Fisher Scientific, Waltham, MA, USA) and acid guanidinium thiocyanate-phenol-chloroform extraction [39]. The isolated RNA was treated with deoxyribonuclease I (DNase I) (Thermo Fisher Scientific) in the presence of RiboLock ribonuclease (RNase) inhibitor (Thermo Fisher Scientific), according to the manufacturer’s recommended protocol. Deproteinization was performed using a 1:1 phenol:chloroform mixture. The isolated RNA was precipitated with sodium acetate (3.0 M, pH 5.2) and ethanol. RNA integrity was checked using capillary electrophoresis (Experion, BioRad, Hercules, CA, USA). RNA integrity number (RIN) was at least 9.0.

### 2.6. RNA-Seq

Total RNA isolated from the subcortical structures of the brain, including the lesion focus, was used in this experiment. The RNA-Seq experiment was conducted with the participation of ZAO Genoanalytika, Russia. For RNA-Seq, the polyA fraction of the total RNA was obtained using the oligoT magnetic beads of the Dynabeads® mRNA Purification Kit (Ambion, Thermo Fisher Scientific, Waltham, MA, USA). cDNA (DNA complementary to RNA) libraries were prepared using the NEBNext® mRNA Library Prep Reagent Set (NEB, Ipswich, MA, USA). The concentration of cDNA libraries was measured using Qbit 2.0 and the Qubit dsDNA HS Assay Kit (Thermo Fisher Scientific, Waltham, MA, USA). The length distribution of library fragments was determined using the Agilent High Sensitivity DNA Kit (Agilent, Lexington, MA, USA). Sequencing was carried out using an Illumina HiSeq 1500 instrument. At least 10 million reads (1/50 nt) were generated.

### 2.7. RNA-Seq Data Analysis

Three pairwise comparisons of RNA-Seq results (IS4.5 versus IR4.5, IS24 versus IR24, and IS24 versus IS4.5) were used to analyse the action of the peptide Semax on the transcriptome. Each of the comparison groups (IS4.5, IS24, IR4.5 and IR24) included three animals (*n* = 3). According to the MRI data (DWI, T2 WI), the ischaemic foci in the brain of these animals had a subcortical localization. 3D-TOF MRA was used for visualization of the main arteries and also for the control of the recanalization. All genes were annotated on the NCBI Reference Sequence database. The levels of mRNA expression were measured as fragments per kilobase per million reads using the Cuffdiff program. Only genes that exhibited changes in expression >1.5-fold and had a *p*-values (*t*-test) adjusted using the Benjamini–Hochberg procedure lower 0.05 (*Pagj* < 0.05) were considered.

### 2.8. cDNA Synthesis

cDNA synthesis was conducted in 20 μL of reaction mixture containing 2 mg of RNA using the reagents of a RevertAid First Strand cDNA Synthesis Kit (Thermo Fisher Scientific, Waltham, MA, USA) in accordance with the manufacturer’s instructions. Oligo (dT)_18_ primers were used to analyse mRNA.

### 2.9. Real-Time Reverse Transcription Polymerase Chain Reaction (RT–PCR)

The 25 μL polymerase chain reaction (PCR) mixture contained 2 μL of 0.2× reverse transcriptase reaction sample, forward and reverse primers (5 pmol each), 5 μL of 5× reaction mixture (Evrogen Joint Stock Company, Moscow, Russia) including PCR buffer, Taq DNA polymerase, deoxyribonucleoside triphosphates (dNTP) and the intercalating dye SYBR Green I. Primers specific to the genes studied were selected using OLIGO Primer Analysis Software version 6.31 (Molecular Biology Insights, Inc., Cascade, CO, USA) and were synthesized by the Evrogen Joint Stock Company, Moscow, Russia (Supplementary Table S1). The amplification of cDNAs was performed using a StepOnePlus Real-Time PCR System (Applied Biosystems, Thermo Fisher Scientific, Waltham, Massachusetts, USA) in the following mode: stage 1 (denaturation), 95 °C, 10 min; stage 2 (amplification with fluorescence measured), 95 °C, 1 min; 65 °C, 1 min; 72 °C, 1 min (40 cycles).

### 2.10. Data Analysis of Real-Time RT–PCR and Statistics

Two reference genes *Gapdh* and *Rpl3* were used to normalize the cDNA samples [40]. Calculations were performed using BestKeeper, version 1 (gene-quantification, Freising-Weihenstephan, Bavaria, Germany) [41] and Relative Expression Software Tool (REST) 2005 software (gene-quantification, Freising-Weihenstephan, Bavaria, Germany) [42]. The manual at the site ‘REST.-gene-quantification.info’ was used to evaluate expression target genes relative to the expression levels of the reference genes. The values were calculated as Ef^Ct(ref)^/Ef^Ct(tar)^, where Ef is the PCR efficiency, Ct(tar) is the average threshold cycle (Ct) of the target gene, Ct(ref) is the average Ct of the reference gene, and Ef^Ct(ref)^ is the geometric average Ef^Ct^ of the reference genes. PCR efficiencies were assessed using the amplification of a series of standard dilutions of cDNAs and computed using REST software [42]. The efficiency values for all PCR reactions were in the range 1.83 to 2.08 (Supplementary Table S1). At least five animals were included in each comparison group (n ≥ 5). When comparing data groups, statistically significant differences were considered with the probability *P* < 0.05. Additional calculations were performed using Microsoft Excel (Microsoft Office 2010, Microsoft, Redmond, WA, USA).

### 2.11. Functional Analysis

Database for Annotation, Visualization and Integrated Discovery (DAVID v6.8, Laboratory of Human Retrovirology and Immunoinformatics, Frederick, MD, USA) [43], Gene Set Enrichment Analysis (GSEA) [44], gProfileR [45] and The PANTHER database (Protein ANalysis THrough Evolutionary Relationships) [46] were used to annotate the functions of the differentially expressed genes. Only functional annotations that had a *p*-values adjusted using the Benjamini–Hochberg procedure lower 0.05 (*Pagj* < 0.05) were considered. Hierarchical cluster analysis of DEGs was performed using Heatmapper (Wishart Research Group, University of Alberta, Ottawa, Canada) [47]. Volcano plot were constructed by Microsoft Excel (Microsoft Office 2010, Microsoft, Redmond, WA, USA).

### 2.12. Availability of Data and Material

RNA-sequencing data have been deposited in the Sequence Read Archive database under accession code SRP148632 (SAMN09235828-SAMN09235839) [48], and PRJNA491404 (SAMN10077190-SAMN10077195) [49].

### 2.13. Ethics Approval and Consent to Participate

All manipulations with experimental animals were approved by the Animal Care Committee of the Pirogov Russian National Research Medical University (Approved ID: 15-2015, 2 November 2015) and were carried out in accordance with the Directive 2010/63/EU of the European Parliament and the Council of European Union on the protection of animals used for scientific purposes issued on 22 September 2010. 

## 3. Results

### 3.1. Characterization of tMCAO Model Conditions Using MRI

The location of ischaemic foci was detected in animals under tMCAO conditions using the T2-weighted imaging (T2 WI) and diffusion-weighted imaging (DWI) of magnetic resonance imaging (MRI). According to MRI, animals after tMCAO under the influence of saline and Semax had the ischaemic zone that was localized in the subcortical structures of the brain from the side of occlusion or spread to the cortex (Supplementary Table S2). Figure S1 shows the MRI of ischaemic foci with a subcortical localization at 4.5 and 24 h after tMCAO under the influence of saline or Semax and MRI of rat brain after sham operation (Supplementary Figure S1).

### 3.2. Histopathological Characterization of Rat Brain

**Figure 1:**
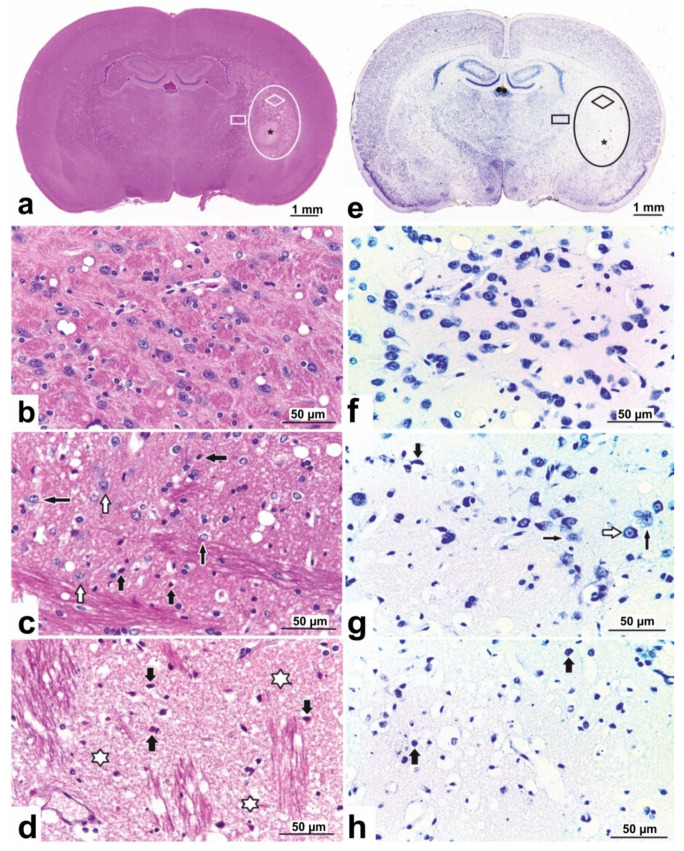
Photomicrographs of haematoxylin and eosin-stained (**a**–**d**) and Nissl-stained (**e**–**h**) sections of the rat brain in tMCAO model conditions. (**a**,**e**) Serial coronal rat brain sections at the level of −2.0 mm from the bregma. Rectangles indicate the normal tissue in the medial region of the caudoputamen. The oval indicates the damaged area involving the lateral region of the caudoputamen nucleus of the right hemisphere. Asterisks indicate necrotic tissue in the central core of an infarct. (**b**,**f**) High-magnification images of the normal tissue in the areas of panels (**a**,**e**) marked with a rectangle. (**c**,**g**) Areas of panels (**a**,**e**) marked with a rhomb. Hypoxic damage to neurons, with pyknotic nuclei and pericellular oedema indicated in the ischaemic zone (thick black arrows); decrease of nuclear basophilia and Nissl substance in the neurons (thin black arrows); intact neurons (white arrows). (**d**,**h**) Areas of panels (**a**,**e**) marked with asterisks. Ischaemic necrosis of the brain tissue in the central core of an infarct; destruction of the neuropil (white asterisks); dead “pyknotic” neurons (thick black arrows), Nissl substance disappearing.

Twenty-four hours after tMCAO, no pathomorphological changes were observed in the cortex of the cerebral hemispheres or the medial region of the subcortical nuclei (Figure 1a,b). After Nissl staining there were no changes in the cytoarchitectonics of the layers of neurons on the cortex of the cerebral hemispheres (Figure 1e). Groups of morphologically unchanged neurons with a basophilic substance in their cytoplasm were located in the medial caudoputamen zone (Figure 1f). However, in the lateral areas of the subcortical region of the right hemisphere, the extensive ischaemic damage to brain tissue was noted as oedema and reticular foci of pale stained foci (Figure 1a,e). In the histological specimens stained with haematoxylin and eosin (H&E), in the caudorostral range from −2.0 to −0.3 mm from the bregma, the ischaemic stroke formation zone captured most of the striatum to the outer capsule with a clear visualization of the nucleus infarction and had an elongated shape in the dorsoventral direction.

On microscopic examination of the penumbra, we found narrowing of the capillary lumen, pronounced perivascular oedema, sparsity and weak staining of the neuropil due to sponginess and vacuolization, and the appearance of numerous hyperchromic neurons with with pyknotic nuclei and pericellular oedema (Figure 1c). A significant part of the neurons was in a state of hypoxic damage or death. In addition, in the ischaemic zones there was a decrease in the content of Nissl-positive neurons, both with partial and total chromatolysis (Figure 1g). Only a few neurons did not have obvious pathological changes.

The nucleus of the infarction was represented by a complete loss of nervous tissue with destruction of pyknotic neurons and all elements of the neuropil (Figure 1d), with Nissl substance disappearing in their perikaryon (Figure 1h). 

In the contralateral hemisphere of ischaemic rats (Figure 1), and in both hemisphere of sham-operated rats (Supplementary Figure S2), the histological pattern of the capillary network and the morphology of neurons in the brain cortex, subcortical nuclei and the intermediate brain corresponded to the norm.

### 3.3. RNA-Seq Analysis of the Effect of Semax on the Transcriptome after tMCAO

**Figure 2:**
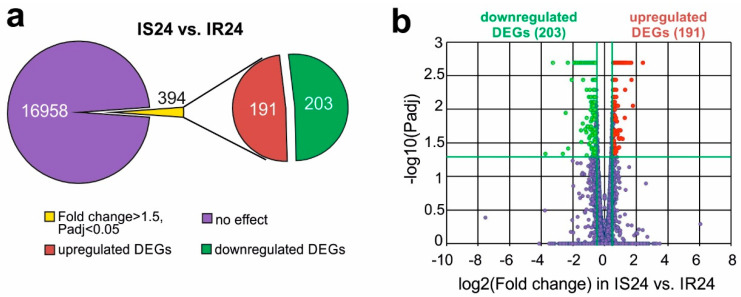
RNA-Seq analysis of the effect of Semax on the transcriptome at 24 h after tMCAO. (**a**) RNA-Seq results presented are for in IS24 versus IR24. The numbers in the diagram sectors indicate the number of DEGs. (**b**) Volcano plots show the distributions of genes between the IS24 and IR24 groups. Up- and downregulated DEGs are represented as red and green dots, respectively (fold change > 1.50. *Padj* < 0.05). Not differential expressed genes are represented as dark purple dots (fold change ≤ 1.50. *Padj* ≥ 0.05).

Using RNA-seq we assessed the effect of Semax on the mRNA level of genes functioning in the subcortical structures of the rat brain at 4.5 and 24 h after tMCAO. Animals subjected to ischaemia–reperfusion (IR) that received saline were used as controls for animals after Semax administration in a tMCAO model at 4.5 h (IS4.5 versus IR4.5) and 24 h after operation (IS24 versus IR24), respectively. From more than 17,000 genes, no significant DEGs (>1.5-fold) were seen after Semax administration versus saline in IR conditions at 4.5 h after tMCAO, but we identified 394 (191 up- and 203 downregulated) DEGs under the influence of Semax at 24 h after tMCAO (IS24 versus IR24) (Figure 2a). The volcano plot shows the up- and downregulated DEGs in IS24 versus IR24 groups (Figure 2b).

We used the real-time RT–PCR analysis of the expression of 4 up- (*Cplx2*, *Gabra5*, *Neurod6*, *Ptk2b*), 4 down- (*Hspb1*, *Hspa1(a,b)*, *Fos*, *Lrg1*) and 2 non-significantly (*Ttr*, *Vegfa*) regulated genes to verify the RNA-Seq results (Supplementary Figure S3). The real-time RT–PCR results confirmed the RNA-Seq data adequately. The differences in the methodology and statistical processing of data used in each case obviously could contribute to some differences in the level of mRNA expression identified by these methods.

### 3.4. Functional Annotation of Semax-Induced DEGs Identified in the Rat Brain Subcortex at 24 h after tMCAO

Using the PANTHER tool (version 15.0, Free Software Foundation, Inc., Boston, MA, USA) we classified Semax-induced DEGs according to the molecular functions of their encoded proteins. In IS24 versus IR24, upregulated DEGs were predominantly associated with receptor activity, signal transducer activity and transporter activity, whereas downregulated DEGs were associated with structural molecule activity, and binding and catalytic activity (Supplementary Table S3).

Using the DAVID program, the functional categories of the proteins encoded by the DEGs were identified (Supplementary Table S4). The total number of them associated with the effects of Semax at 24 h after tMCAO (IS24 versus IR24) was 34. The top 5 functional categories with the smallest *Padj* were Disulfide bond, Glycoprotein, Signal, Secreted and Calcium. Functional categories of neurotransmission systems of cells (Synapse, Postsynaptic cell membrane, Calmodulin-binding, and Cell junction) were associated predominantly with upregulated DEGs in IS24 versus IR24 (Supplementary Table S4). Simultaneously, functional categories of immunity and inflammatory responses (Immunity, Intermediate filament, and Innate immunity) were associated predominantly with downregulated DEGs in IS24 versus IR24 (Supplementary Table S4).

### 3.5. Differences in Rat Brain Transcriptomes Following Ischaemia and after Semax Administration

**Figure 3:**
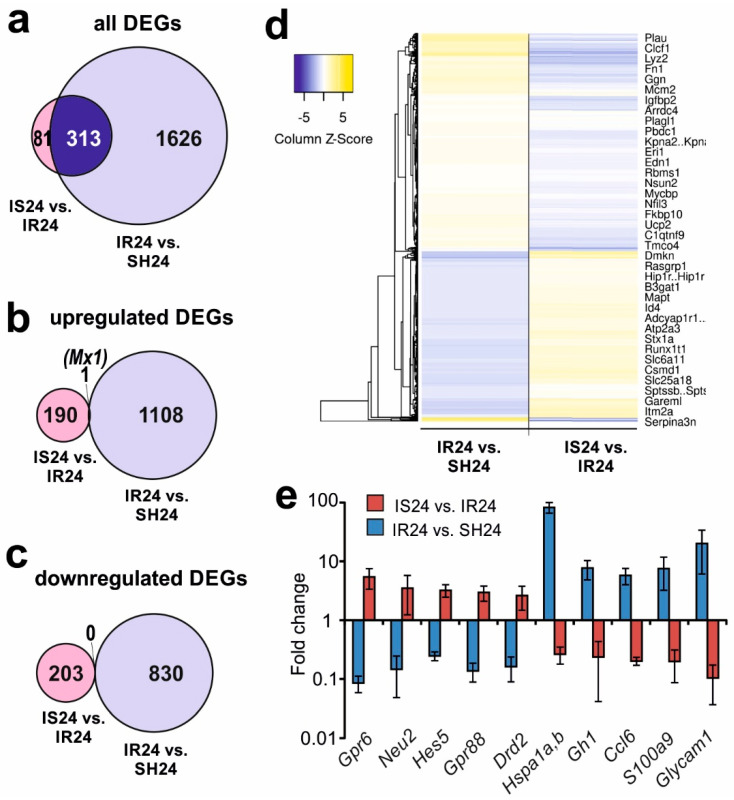
Comparison of the results obtained in two pairwise comparisons of IS24 versus IR24 and IR24 versus SH24. (**a**–**c**) Venn diagrams of DEGs in two pairwise comparisons of IS24 versus IR24 and IR24 versus SH24. Comparison for all (**a**), up- (**b**) and downregulated (**c**) DEGs. (**d**) Hierarchical cluster analysis of all DEGs in IS24 versus IR24 and IR24 versus SH24. Each column represents a comparison group, and each row represents a DEG. Yellow strips represent high relative expressions and blue strips represent low relative expressions, *n* = 3 per group. (**e**) The top ten genes that exhibited the greatest fold change in expression in IS24 versus IR24. The data are presented as the mean ± standard error of the mean. The cut-off of gene-expression changes was 1.50. Only those genes, whose *Padj* < 0.05 were selected for analysis.

Previously, using RNA-Seq, we identified 1939 DEGs (1109 up- and 839 downregulated) under IR conditions after tMCAO at 24 h versus sham operation at 24 h after surgical procedure (IR24 versus SH24) [32]. In this study, we identified 313 DEGs that overlapped for IS24 versus IR24 and IR24 versus SH24 (Figure 3a, Supplementary Table S5). Venn diagrams with only up-regulated DEGs and only down-regulated DEGs in both conditions are shown in Figure 3b,c. We found only the *Mx1* gene, which encodes an interferon-induced GTP-binding protein was upregulated in both IS24 versus IR24 and IR24 versus SH24, simultaneously (Figure 3b), and did not found DEGs which were downregulated in both conditions (Figure 3c). It should be noted that Semax initiated mRNA expression that counteracted the effects of IR injury. Hierarchical cluster analysis shows the Semax increases the expression levels of genes that reduce expression by the action of ischaemia and vice versa, that is, it compensates for the effect of ischaemia (Figure 3d). In particular, we found 155 up- and 157 downregulated DEGs in IS24 versus IR24 that counteracted IR in IR24 versus SH24 (Supplementary Table S5). The top 10 genes with the greatest fold change in IS24 versus IR24 are presented in Figure 3e. The figure shows that the expression levels of genes *Gpr6*, *Neu2*, *Hes5*, *Gpr88*, and *Drd2* was increased by up to 2.5-fold in IS24 versus IR24 and was decreased by up to 4-fold in IR24 versus SH24. Simultaneously, the expression of genes *Glycam1*, *S100a9*, *Ccl6*, *Gh1*, and *Hspa1(a,b)* was decreased by 3-fold in IS24 versus IR24 and was increased by up to 5-fold in IR24 versus SH24.

Moreover, 81 genes (35 up- and 46 downregulated) altered their expression in IS24 versus IR24 but did not alter it in IR24 versus SH24 (Figure 3a, Supplementary Table S6). These DEGs predominantly have catalytic and binding activity, but additionally have receptor and signal transducer (*Gabrb1*, *Grin2b*, *Folr1*, *Bdnf*), as well as transporter (*Kcnj13*, *Slc1a2*, *Slco1a5*) activity (Supplementary Table S7).

### 3.6. Signalling Pathways Associated with Semax-Induced DEGs under tMCAO Model Conditions

**Figure 4:**
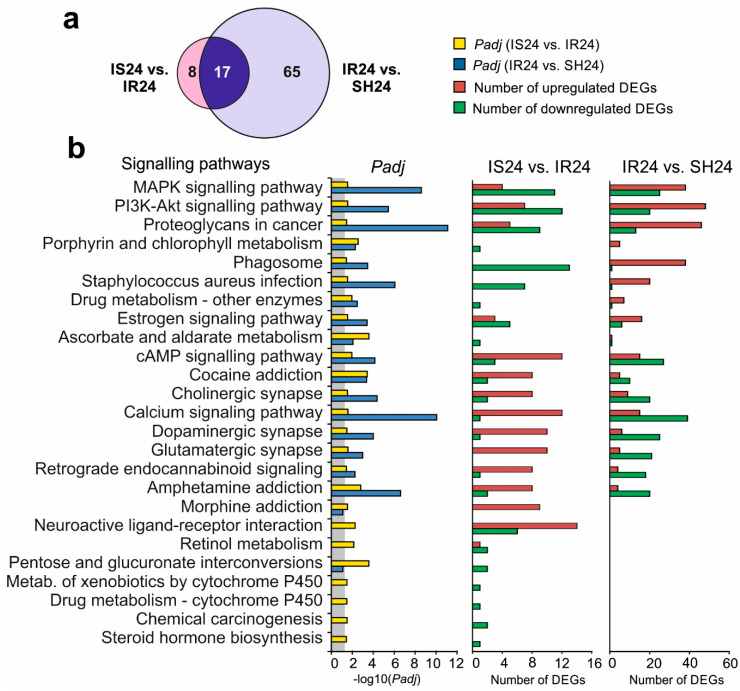
Analysis of the signalling pathways associated with DEGs at 24 h after tMCAO. (**a**) The numbers of signalling pathways overlapped in two pairwise comparisons: IS24 versus IR24 and IR24 versus SH24. (**b**) KEGG database analyses of DEGs in two pairwise comparisons IS24 versus IR24 and IR24 versus SH24 was carried out according to the DAVID database. The number of upregulated and downregulated DEGs, as well as the *p*-values adjusted using the Benjamini–Hochberg procedure (*Padj*), are shown. Only those genes and signalling pathways whose *Padj* < 0.05 were selected for analysis. *Padj* ≥ 0.05 are enclosed in the gray background.

Semax-induced DEGs identified in the rat brain subcortex under tMCAO model conditions were annotated according to a Kyoto Encyclopedia of Genes and Genomes (KEGG) using DAVID v6.8. We found 25 signalling pathways associated with DEGs in IS24 versus IR24. Previously, we found 82 signalling pathways associated with DEGs detected in the brains of rats in the IR24 versus SH24 groups [32]. Among them, 17 signalling pathways overlapped between IS24 versus IR24 and IR24 versus SH24 (Figure 4a). These signalling pathways are involved in neurotransmission, drug metabolism and inflammation. There were 8 signalling pathways (Porphyrin and chlorophyll metabolism, Drug metabolism—other enzymes, Phagosome, PI3K-Akt and MAPK and other signalling pathways) predominantly associated with the upregulated DEGs in IR24 versus SH24 and downregulated DEGs in IS24 versus IR24 (Figure 4b). Conversely, there were 8 signalling pathways (Amphetamine addiction, Retrograde endocannabinoid signalling, Glutamatergic and Dopaminergic synapses and other signalling pathways) predominantly associated with the downregulated DEGs in IR24 versus SH24 and upregulated DEGs in IS24 versus IR24. Additionally, similar results were obtained using GSEA (KEGG, Reactome, BioCarta, PID) and gProfileR (KEGG, Reactome, WikiPathways). So, based on GSEA data, upregulated DEGs in IS24 versus IR24 were associated with Neuronal System, Signaling by G-protein-coupled receptors (GPCR), Transmission across Chemical Synapses, etc; whereas downregulated DEGs were associated with Innate Immune System, Neutrophil degranulation, Cytokine Signalling in Immune system, etc (Supplementary Table S8). Also, based on gProfileR data, upregulated DEGs in IS24 versus IR24 were associated with, Calcium signaling pathway, Dopaminergic, Cholinergic and Glutamatergic synapse, etc; whereas downregulated DEGs were associated with Phagosome, IL-17, TNF, p53 signaling pathway, etc (Supplementary Table S9). Thus, Semax initiated neurotransmitter and inflammatory response that counteracted IR at 24 h after tMCAO.

## 4. Discussion

Drugs, based on native regulatory peptides, are used to treat various pathological conditions, including peptide drugs that help restore brain function after acute cerebrovascular accidents. In studies to investigate the molecular mechanisms of action of such neuroprotective peptides, experimental models of ischaemia in animals are of great importance. For example, the neuroprotective effects of orexin (OxA) [50] and poly-arginine R18 and NA-1 peptides [51], as well as the anti-inflammatory and anti-oxidant effects of cordymin peptide [52] were identified in studies employing middle cerebral artery occlusion-induced focal cerebral ischaemia in rats. Semax, a nootropic neuropeptide, has been used in neurological practice for many years in the treatment of acute and chronic disorders, including ischaemic stroke and its consequences [14,15,53]. However, its molecular mechanisms of action are not yet fully understood. There are many examples of the use of transcriptome analysis to study the mechanisms of action of a number of peptides including PACAP38 [54], Sal-like 4 peptide [55] and OxA [50]. The first genome-wide analysis of Semax action on the rat brain transcriptome was conducted using a permanent MCAO model that was induced by direct permanent electrical coagulation of the distal segment of the left middle cerebral artery [26,27]. The genome-wide biochip data analysis detected DEGs associated with several biological processes and signalling pathways. In the first hours after pMCAO, a significant increase in the expression of transcription factor genes was observed in the presence of Semax. These genes encode proteins that trigger signalling to correct the destructive processes associated with ischaemic conditions. Semax also stimulated the expression of genes that encode growth factors involved in trophic and protective processes, as well as the vascularization of damaged tissues. Additionally, Semax administration had a significant effect on the expression of genes associated with the immune response [26,27].

In the current study of the protective mechanisms of action of the Semax peptide, we aimed to identify the DEGs, as well as the biological processes and signalling pathways involved in the response of rat brain cells to Semax action under ischaemia–reperfusion (IR) conditions. Therefore, we used the tMCAO model, which involves occlusion of the MCA and subsequent restoration of blood flow [22,56]. Importantly, the tMCAO model in rats replicates circumstances that arise in the human brain after ischaemic stroke treated with thrombolytic medications [28,29]. MRI (Supplementary Table S2 and Figure S1) and histopathological analysis (Figure 1) detected the ischaemic focus and the penumbra region in the subcortical structures of the brain after tMCAO. A study of the molecular mechanisms of cell death under pMCAO and tMCAO conditions, conducted by Ford et al. [57] revealed molecular functions and biological processes unique to each model. Genes uniquely altered by tMCAO included a number of genes related to inflammatory and oxidative stress, whereas pMCAO led to the induction of genes that were more associated with metabolic activity and cellular signalling [57]. In addition, reperfusion after ischaemia leads to additional injury, including disturbance of the blood-brain barrier via destruction of the endothelial microvascular brain cells, and damage of brain cells through the accumulation of excess oxygen radicals and apoptosis [22,58,59,60,61].

Using RNA-Seq to determine the mRNA expression profile in the subcortical structures of rat brain in response to Semax administration, a significant effect of Semax was identified at 24 h after tMCAO. We identified 394 DEGs (>1.5-fold change) following Semax administration at 24 h after tMCAO, but there were no DEGs identified following Semax administration at 4.5 h after tMCAO. An analysis of the expression of 10 genes by real-time RT-PCR confirmed the RNA-Seq results. A large number of genes, altered by Semax in the tMCAO model, encode transcripts and proteins involved in cellular processes, metabolic processes, biological regulation, responses to external stimuli and multicellular organism-associated processes. 

In a previous study, we revealed the large transcriptome response of cells in the subcortical structures of rat brain at 24 h after tMCAO and identified the biological processes and signalling pathways involved in the response to IR damage [32]. These conditions produced activation of a large number of genes involved in inflammation, the immune response, apoptosis, stress responses, ribosome function, DNA replication and other processes (e.g., *Hspa1(a,b)*, *Hspb1*, *Lrg1*, *Jun*, *Socs3*, *Cish*, *Cd14*, *Cd63*, *Cd74*, *Ccl6*, *Ccl9*, *Nfkb2*, *Fos* and others). In contrast, the expression of many genes related to neurotransmitter system function was inhibited (e.g., *Chrm1*, *Chrm4*, *Cplx2*, *Drd1*, *Drd2*, *Gabra5*, *Gria3*, *Grm3*, *Grm5*, *Gpr6*, *Gpr88*, *Htr6*, *Neurod6* and others) [32].

In the current study, we first compared the transcriptome response of brain cells to IR (IR24 versus SH24) and the effect of Semax treatment under IR conditions (IS24 versus IR24). These analyses revealed that the effect of the peptide on several hundred genes was opposite to that of IR damage. Under IR conditions, Semax administration upregulated the expression of *Gpr6*, *Neu2*, *Hes5*, *Gpr88*, *Drd2* and others, the genes predominantly involved in the neuronal receptor actions and neurogenesis, and downregulated the expression of chemokine genes (*Ccl6*, *Ccl9*), early response genes (*Hspa1(a,b)*, *Fos*, *Jun*) and others (Figure 3e). These data suggest that Semax can normalize the expression of many genes disrupted during IR.

The functional annotation of Semax-induced DEGs under tMCAO model conditions also demonstrated the compensatory effect of the peptide on the functioning of a number of signalling and metabolic systems after IR. Semax suppressed the inflammatory, and activated the neurotransmitter signalling pathways, at 24 h after tMCAO (Figure 4), whereas we previously revealed that IR activated inflammatory, but suppressed neurotransmitter signalling pathways at 24 h after tMCAO [32]. Therefore, Semax initiated genetic responses that counteracted those produced by IR injury.

Our data on the activation of neurotransmitter signalling pathways by Semax are in good agreement with previous research, which reported a positive modulatory effect of Semax on the striatal serotonergic system and the ability of Semax to enhance the striatal release of dopamine [62]. Semax has also been shown to modulate GABA- and glycine-activated ionic currents in isolated cerebral neurons [63]. Earlier it was shown that Semax (1 μM) did not affect cyclic adenosine monophosphate (cAMP) levels in HEK293 cells expressing the human melanocortin 4 receptor (MCR4), but antagonized the cAMP-inducing effect of the melanocortin derivative α-melanocyte-stimulating hormone (α-MSH) in this cells [64]. Additionally, in models of brain ischaemia and inflammation, α-MSH exhibits strong anti-inflammatory, neurogenic and neuroprotective effects [65,66,67]. Therefore, Semax may recapitulate some important neuroprotective actions of the melanocortins.

The major limitation of our study is the lack of data confirming the change in the protein level of mRNA expression of detected DEGs, and any functional evidence supporting the change in the mRNA expression of these genes. In the future, further studies on the functioning of a limited number of key genes of the detected signaling pathways at the RNA and protein levels are necessary to be done. However, the main achievement is the full transcriptome study with the identification of specific signaling pathways involved in the molecular mechanisms of the peptide action. Bulk RNA-seq, in combination with bioinformatic approaches, remain dominant and valuable tools for the simultaneous analysis of changes in mRNA expression of a huge number of genes and the search for signaling pathways that are involved in the response to specific exposure. It was precisely the analysis of RNA-seq that allowed us to establish that the Semax peptide suppresses the expression of genes associated with inflammatory processes and activates the expression of genes related to neurotransmission. Significantly, these transcriptome changes driven by Semax are the opposite of those caused by IR.

In summary, our data indicate that an important feature of the neuroprotective action of Semax is the normalization of mRNA expression patterns that are disrupted during IR conditions, particularly those associated with anti-inflammatory processes and activation of neurotransmitter systems.

## 5. Conclusions

In conclusion, the study of the transcriptome profile of cells in the subcortical structures of the brain with administration of the neuropeptide drug Semax under tMCAO conditions led to the identification of DEGs that encode proteins that participate in various functional categories, biological processes and signalling pathways, via which brain cells form a response to ischaemia–reperfusion (IR). Under tMCAO conditions, we found that Semax initiated mRNA expression that counteracted IR. In particular, Semax suppressed inflammatory and activated neurotransmitter genes, whereas the genetic response initiated by IR activates inflammatory and suppresses neurotransmitter genes. We revealed significant compensation effects of Semax peptide on inflammatory and neurotransmitter genetic responses after tMCAO, which may account for the neuroprotective action of Semax under IR conditions. Thus, an important feature of Semax is the normalization of mRNA expression patterns that are disturbed during ischaemia.

## Supplementary Materials

The following are available online at https://www.mdpi.com/2073-4425/11/6/681/s1, Figure S1: Characterization of tMCAO model conditions using MRI, Figure S2: Morphology of brain tissues after sham operation, Figure S3: Real-time reverse transcription polymerase chain reaction (RT-PCR) verification of the RNA-Seq results, Table S1: The characterization of the primers for real-time RT-PCR, Table S2. Quantitative assessment of the volume of the ischemic brain injury at 24 hours after tMCAO using magnetic resonance imaging, Table S3: Analysis of the molecular function associated with Semax-induced DEGs (IS24 versus IR24) using the PANTHER tool, Table S4: Analysis of the functional categories of the proteins encoded by DEGs in IS24 versus IR24, Table S5: Analysis of DEGs that overlapped between IS24 versus IR24 and IR24 versus SH24, Table S6: Analysis of DEGs that changed their expression in IS24 versus IR24, but did not change it in IR24 versus SH24, Table S7: Analysis of the molecular functions associated with the DEGs using the PANTHER tool, Table S8: Analysis of the functional annotations of DEGs in IS24 versus IR24 using GSEA, Table S9: Analysis of the functional annotations of DEGs in IS24 versus IR24 using gProfileR.
