# Supplementary material for: Novel Insights into the Protective Properties of ACTH(4-7)PGP (Semax) Peptide at the Transcriptome Level Following Cerebral Ischaemia–Reperfusion in Rats
Source: Genes (Basel). 2020 Jun 22;11(6):681. doi: 10.3390/genes11060681 (PMC7350263; doi:10.3390/genes11060681)
Supplement: Supplementary file 1 [file genes-11-00681-s001.zip › Supplementary Figure S3.docx]

**Supplementary Figure S3. Real-time reverse transcription polymerase chain reaction (RT-PCR) verification of the RNA-Seq results.**


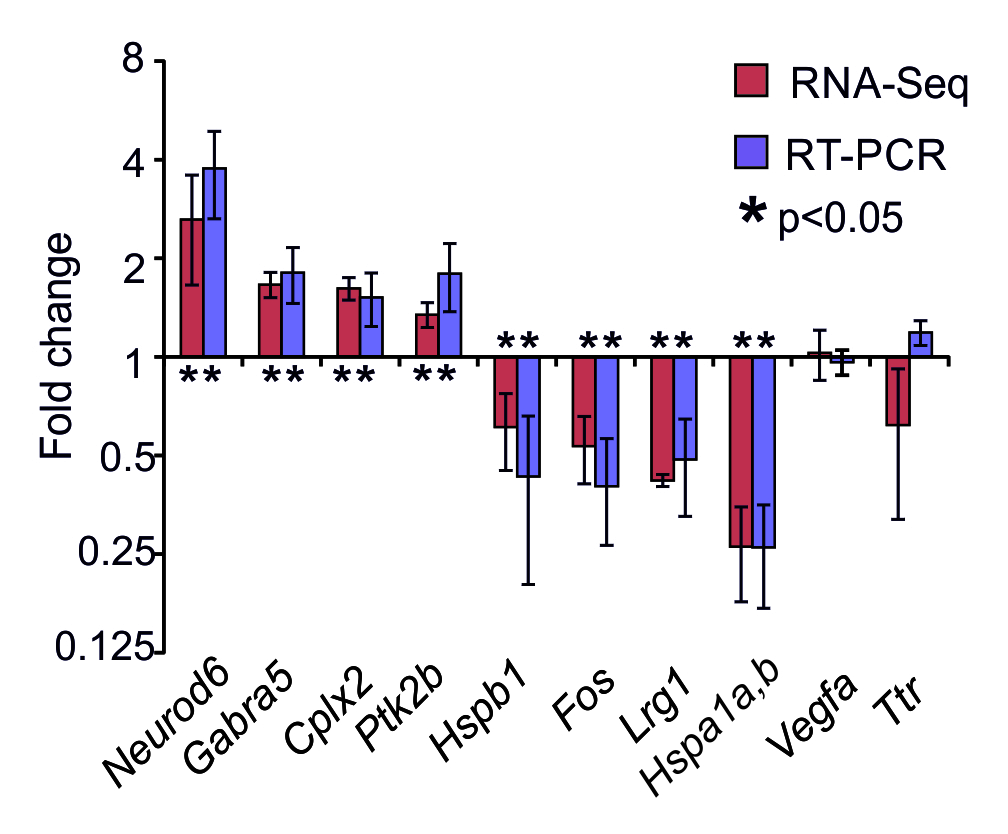


Data for comparison in IS24 vs. IR24 are shown. Two reference genes *Gapdh* and *Rpl3* were used to normalize PCR-results. Eight genes, whose *P*-value lower 0.05, as well as two other genes were selected for analysis. The data are presented as the mean ± standard error of the mean.
