# Supplementary material for: Novel Insights into the Protective Properties of ACTH(4-7)PGP (Semax) Peptide at the Transcriptome Level Following Cerebral Ischaemia–Reperfusion in Rats
Source: Genes (Basel). 2020 Jun 22;11(6):681. doi: 10.3390/genes11060681 (PMC7350263; doi:10.3390/genes11060681)
Supplement: Supplementary file 1 [file genes-11-00681-s001.zip › Supplementary Figure S1.pdf]

Supplementary Figure S1

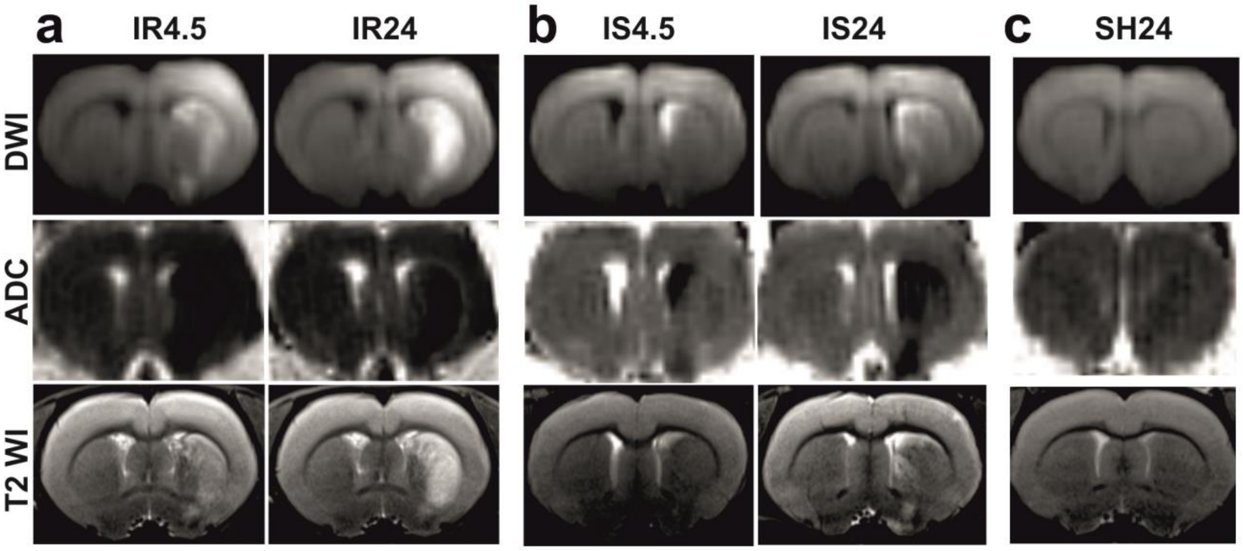

Figure S1. Characterization of tMCAO model conditions using MRI. (**a, b**) MRI of ischaemic foci at 4.5 and 24 h after tMCAO. DWI with an ADC map and T2 WI scans of the formation of ischaemic injury areas with a subcortical localization in the brain of rats after saline administration at 4.5 (IR4.5) and 24 h (IR24) after tMCAO (**a**) and after Semax administration at 4.5 (IS4.5) and 24 h (IS24) after tMCAO (**b**). (**c**) DWI with an ADC map and T2 WI scans of the brain of rats at 24 h after sham operation (SH24).
