# Supplementary material for: Novel Insights into the Protective Properties of ACTH(4-7)PGP (Semax) Peptide at the Transcriptome Level Following Cerebral Ischaemia–Reperfusion in Rats
Source: Genes (Basel). 2020 Jun 22;11(6):681. doi: 10.3390/genes11060681 (PMC7350263; doi:10.3390/genes11060681)
Supplement: Supplementary file 1 [file genes-11-00681-s001.zip › Supplementary Figure S2.pdf]

Supplementary Figure S2

Morphology of brain tissues after sham operation

Microscopic examination of the brain of sham-operated animals shows a histological picture of the cerebral cortex (Figure S2a, b) and neurogenesis in the rostral subventricular zone (Figure S2c) corresponded to the norm for the given species and age of the animals. No pathological changes were detected in the neurons of the cortex (Figure S2b), subcortical nuclei (Figure S2c) and thalamus (Figure S2d).

At Nissl-stained specimens, the cytoarchitectonics of the cortex hemispheria cerebri in the layers of neurons does not reveal any specific findings (Figure S2e, f). In the hippocampus, neurons of the granular layer of the dentate gyrus (Figure S2g) and the pyramid layers in the CA1-CA3 fields were well structured, had a moderately basophilic cytoplasm and a rounded nucleus with granular chromatin and a small nucleolus. Nissl's basophilic substance was clearly visualized in the perikaryon of the thalamic neurons (Figure S2h).

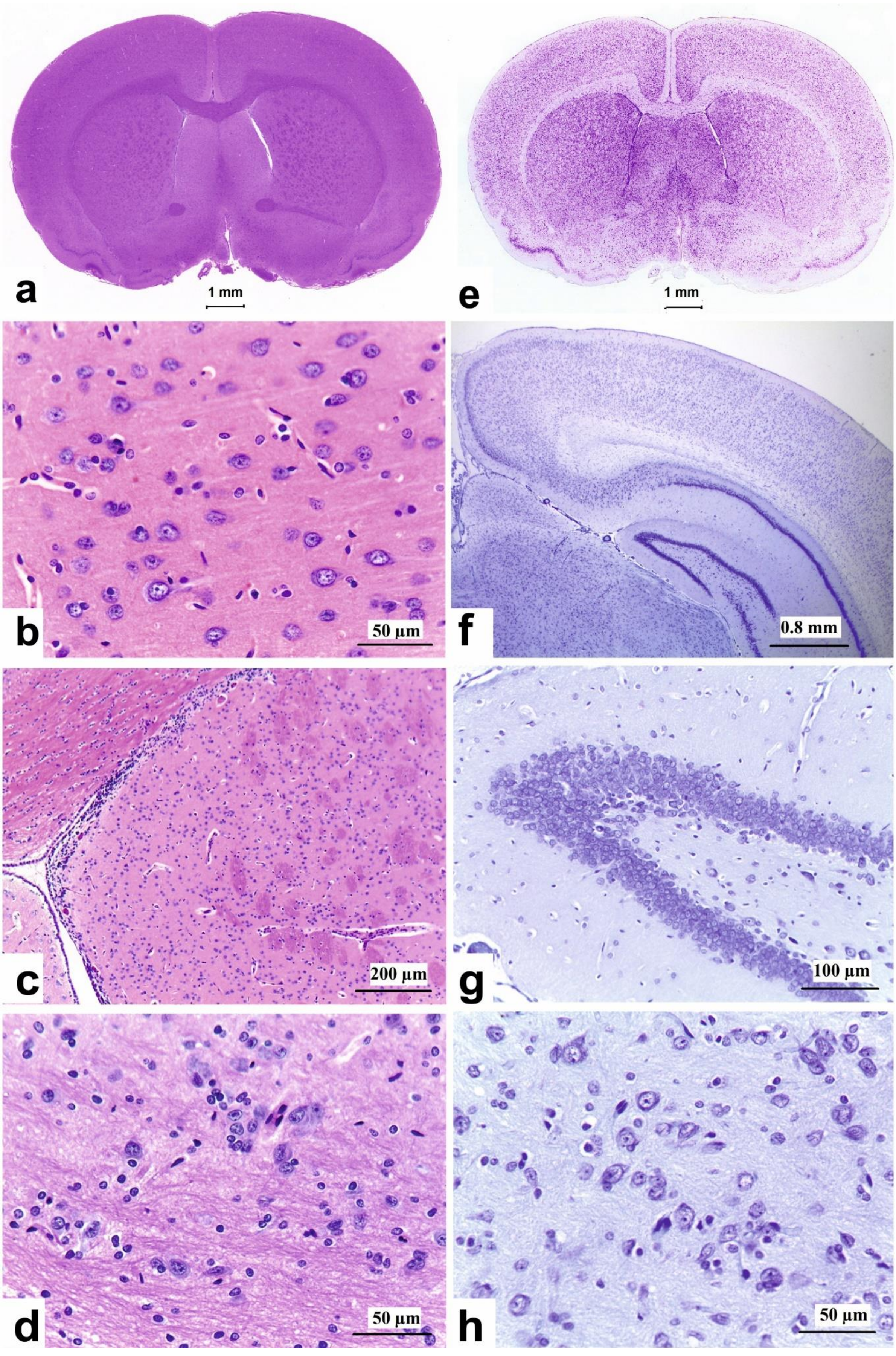

Figure S2. Photomicrographs of haematoxylin and eosin-stained (**a**–**d**) and Nissl-stained (**e**–**h**) sections of the rat brain of sham-operated rat. (**a, e**) Serial coronal rat brain sections at the level of +0.7 mm from the bregma. (**b**) Neurons of the pyramidal layer of the cortex of the right hemisphere. (**c**) Neurogenesis zone

and medial caudoputamen region of the right hemisphere. (**d**) Neurons in the thalamus. (**f**) The histostructure of the dorsolateral region of the right hemisphere at the hippocampus (the level of -3.3 mm from the bregma). (**g**) Neurons of the granular layer of the dentate gyrus of the hippocampus (fragment of Figure “f” at higher magnification). (**h**) Thalamic neurons.
